# Personality and intentional binding: an exploratory study using the narcissistic personality inventory

**DOI:** 10.3389/fnhum.2015.00013

**Published:** 2015-02-05

**Authors:** Ann (Chen) Hascalovitz, Sukhvinder S. Obhi

**Affiliations:** ^1^Department of Physiology, Development and Neuroscience, Behavioural and Clinical Neuroscience Institute, University of CambridgeCambridge, UK; ^2^Social Brain, Body and Action Lab, Department of Psychology, Neuroscience and Behaviour, McMaster UniversityHamilton, ON, Canada

**Keywords:** intentional binding, agency, narcissism, narcissism and agency, narcissism and intentional binding, subjective time, awareness of action

## Abstract

When an individual estimates the temporal interval between a voluntary action and a consequent effect, their estimates are shorter than the real duration. This perceived shortening has been termed “intentional binding”, and is often due to a shift in the perception of a voluntary action forward towards the effect and a shift in the perception of the effect back towards the action. Despite much work on binding, there is virtually no consideration of individual/personality differences and how they affect it. Narcissism is a psychological trait associated with an inflated sense of self, and individuals higher in levels of subclinical narcissism tend to see themselves as highly effective agents. Conversely, lower levels of narcissism may be associated with a reduced sense of agency. In this exploratory study, to assess whether individuals with different scores on a narcissism scale are associated with differences in intentional binding, we compared perceived times of actions and effects (tones) between participants with high, middle, and low scores on the narcissistic personality inventory (NPI). We hypothesized that participants with higher scores would show increased binding compared to participants with lower scores. We found that participants in our middle and high groups showed a similar degree of binding, which was significantly greater than the level of binding shown by participants with the lowest scores. To our knowledge, these results are the first to demonstrate that different scores on a personality scale are associated with changes in the phenomenological experience of action, and therefore underscore the importance of considering individual/personality differences in the study of volition. Our results also reinforce the notion that intentional binding is related to agency experience.

## Introduction

Whereas there is large body of research on the production and control of human action, there is less work devoted to understanding the subjective experience of action (Rosenbaum, 1991; Haggard, 2001; Obhi and Goodale, 2005). The sense of agency refers to the feeling of control over self-produced actions and, as a consequence, the feeling of being a causal agent capable of effecting change in the environment (Gallagher, 2012; Moore and Obhi, 2012). The sense of agency can be either an explicit phenomenologically rich conscious experience of control, or can be relatively phenomenologically “thin”, such as when a person “knows” that they acted to cause some effect, but such knowledge does not become the focus of conscious awareness. Understanding the neurocognitive processes that underlie both forms of agency experience has become an important goal for cognitive neuroscience and experimental psychology.

To the extent that actions produce effects in the environment, they can be considered as operant. From over a decade of research, an important finding is that, when such operant actions are made volitionally, the actor perceives the time interval between the action and the consequent effect to be shorter than its true value (Haggard et al., 2002; see Moore and Obhi, 2012 for a review). More specifically, this illusory interval compression usually manifests as a perception that the initiation of action occurs later than it actually did, and a perception that the effect occurred earlier than it actually did, although in certain cases effects have been found on the percept of one component of the action-effect complex and not the other. Interestingly, if the person is *made* to perform the action (and thus produce the effect) involuntarily, either by transcranial magnetic stimulation (TMS) of the motor cortex, or by other mechanical means, the perceived shortening of the action-effect interval does not occur (Tsakiris and Haggard, 2003). The apparent dependence of this temporal illusion on intention seems to support the notion that the illusion may be linked to the sense of agency. The illusion has thus been referred to as “intentional binding”. The potential link between intentional binding and the sense of agency is intriguing and has spawned considerable interest from researchers in experimental psychology and cognitive neuroscience, although it is noteworthy that some researchers have exercised caution in interpreting the effect in terms of intentional processes and instead consider it as a special case of more general cause-effect processing (Buehner, 2012).

In this light, and in order to better understand whether intentional binding is linked to agency, it is necessary to investigate the conditions under which intentional binding occurs, with specific regard to the personal and situational factors that modulate the magnitude of the effect. This has often been done using experimental manipulations of action-effect contingency which influence the ability to predict the outcome of actions, and has even extended into questions about the moral status of an outcome, joint actions and the effects of recalling memories of power and depression (Moore et al., 2009; Moretto et al., 2011; Obhi and Sebanz, 2011; Obhi et al., 2012, 2013).

Another approach has been to assess binding in patients who are known to have deficits in the production, control and subjective experience of action. In this regard, patients such as those with schizophrenia, Parkinson’s disease and psychogenic conversion disorders have been found to display “abnormal” patterns of binding (Haggard et al., 2003; Kranick et al., 2013). However, despite some limited work in patient populations, to date, there has been no research investigating the effects of variation in specific personality traits on binding. Indeed, more generally, the question of how action is experienced by individuals with different psychological profiles remains largely ignored.

Similar to the patient approach, studying the relationship between personality traits and binding could be useful in shedding light on the purported link between binding and agency. Specifically, individuals who possess traits that are linked to differences in the tendency to act, or to the perception of the self as a powerful entity, might be expected to show differences in binding. In the present study, to further investigate the notion that binding is linked to agency, we contrasted neurologically normal individuals who differ on their scores on the narcissistic personality inventory (NPI), a commonly used index of sub-clinical narcissism in social psychological research. Narcissism is a personality trait that has been linked to an inflated sense of self, a tendency toward high levels of dominance motivation and dominance behavior, and a perception of the self as a powerful agent (Kohut, 1977; Raskin et al., 1991; Morf and Rhodewalt, 2001). Despite this stereotypical view of the powerful and dominant narcissist, it is worth noting that accounts of clinical narcissists often reveal a rather fragile picture in which narcissists are prone to feelings of emptiness, a lack of belonging and fluctuating self-esteem. Indeed, Kohut has argued that behind the grandiosity, lies low self-esteem (Kohut, 1971). Others suggested that narcissists overcome this situation via greater than normal self-enhancement (John and Robins, 1994). In their proposal for an integrated model of narcissism, Dimaggio et al. (2002) observe that narcissists often engage in an ever-escalating process of self-enhancement, which they employ to protect fragile self-esteem. These authors suggest that narcissists do not have the requisite metacognitive skills to understand why they don’t fit in, and they tend to deal with such situations which leave them feeling disconnected and separate, by self-administering self-esteem tests, which they tend to pass due to self-enhancement. This process has been associated with threatening swings in self-esteem, which further contribute to the fragility of the narcissistic mindset (Ronningstam, 2011a; for more on manifestation of clinical narcissism see Dimaggio et al., 2006, 2008). However, individuals with sub-clinical levels of narcissism, do appear to maintain a higher level of self-esteem and self-agency and are therefore somewhat more stable than their clinical coutnerparts (Ackerman et al., 2011). Individuals who score higher on subclinical narcissism have been shown to pursue dominance behaviors in order to maintain their grandiose sense of self, and from an evolutionary perspective, to gain better access to resources via increased social status (Baumeister et al., 2000; Kirkpatrick et al., 2002). Indeed, when scores on measures such as the NPI are examined in relation to scores on self-report measures of agency, there is a strong positive correlation between the two (Campbell et al., 2007). This finding, coupled with the purported link between agency and intentional binding, makes it important to characterize the relationship between narcissism and intentional binding.

In the current study, to shed more light on the link between scores on the NPI, intentional binding and agency, we recruited individuals who had previously completed the NPI. We allocated individuals to a high, middle, and low groups based on the range of NPI scores in our sample and ran each participant through an intentional binding experiment. During this intentional binding task, participants were asked to judge the onset time of actions and consequent effects. The task involved making an action (clicking a mouse), experiencing an effect (hearing a tone), and making an action that resulted in a subsequent effect (clicking the mouse to produce the tone), while watching clock hand rotate on a computer screen (Haggard et al., 2002). During the different conditions, participants reported where the clock hand was when they clicked the mouse or heard the tone. By calculating the difference between the perceived time of the action when it did not produce a tone against when it did produce a tone, and the perceived time of the tone when it was preceded by an action against when it was not preceded by an action, we determined the intentional binding effect. Importantly, intentional binding is thought to represent an implicit measure of the sense of agency (see Moore and Obhi, 2012 for a review). Given the purported link between narcissism and agency, we predicted that individuals with higher NPI scores would demonstrate significantly greater levels of intentional binding compared to those with lower NPI scores.

## Methods

### Participants

Twenty-seven university students (nine males and 18 females, age 17–20, Mean 18.3, SD 0.73) participated in the study for a course credit or $11 compensation. Each participant was run individually in a single cubicle with the researcher present. The participants had all been previously screened online using the NPI and grouped into the high and low narcissism group prior to the experiment based on the distribution of scores on the NPI taken as part of mass testing. Specifically, participants were allocated to the “high” group if their score on the NPI was over 21 out of a possible 40, and participants were allocated to the “low” group if their NPI score was less than 10. The middle group was created based on the distribution of scores between the high and low groups, and was comprised of individuals who scored between 11–17 on the NPI. This resulted in the inclusion of nine participants in each of the three groups. All participants completed a written consent form at the beginning of the study. It is important to note that being placed into the high group does not correspond to being a pathological “narcissist” and we are not making any claims in this paper about narcissistic personality disorder (NPD). Indeed, the label “high” in this paper simply refers to a relatively high score in the range of scores we obtained in the current sample. Our simple aim in this exploratory study was to assess whether there are measurable differences in intentional binding associated with individuals whose score on the NPI differs.

### Apparatus and stimuli

The experiment was programmed using Superlab version 4.5 (Cedrus Corporation, San Pedro, CA, USA) and was run on a Lenovo computer, with stimuli displayed on a 19-inch LCD monitor. A Microsoft serial mouse was used to register the voluntary key press (left click). Auditory tones (100 ms, 1000 Hz, were presented over Dell Desktop speakers situated either side of the computer monitor).

### Procedure

**Figure 1:**
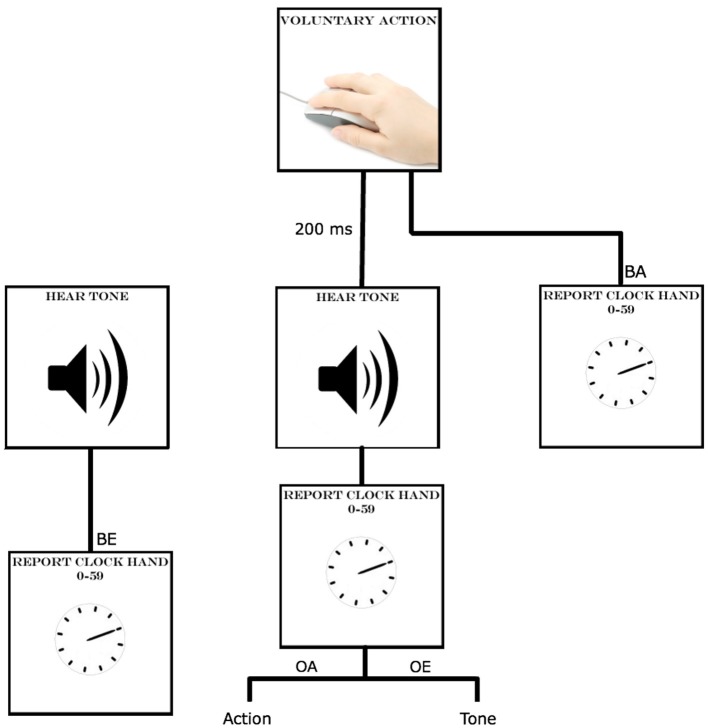
**Procedure for the intentional binding experiment, labeled BA, BE, OA and OE for: baseline action, baseline effect, operant action and operant effect conditions**.

Participants completed the experiment one at a time with the experimenter present in a testing cubicle. Participants were instructed to watch a small clock (2.5 cm diameter, marked at 5 min intervals) rotate on the computer screen and, depending on the condition, to report where the clock hand was when they either pressed the key or heard the tone (between 0 and 59, see Haggard et al., 2002; Obhi and Hall, 2011 for a similar approach). There were four different conditions, or blocks, that each subject completed in a pseudo-random order: baseline action, baseline effect, operant action and operant effect (Figure 1). Each block had 60 trials and clock hand starting position was pseudo-randomly varied. In the baseline action condition, participants were instructed to click the mouse at a time of their own choosing (and not in response to position of the clock hand). After their key press, the clock hand continued to rotate for a variable amount of time. At the end of the trial, participants were asked to report to the researcher where the clock hand was when they initiated their voluntary action. In the baseline effect condition, participants were asked not to produce a key press, but instead watch the clock and report the clock hand position at the time a randomly occurring tone sounded (tone could occur between 1600 and 3600 ms after the appearance of the clock). In the operant action condition, participants were again instructed to click the mouse at a time of their own choosing after the appearance of the clock. Upon clicking the mouse, a tone sounded and, at the end of the trial, participants were asked to report where the clock hand was when they clicked the mouse, not when they heard the tone. Finally, in the operant effect condition, participants again clicked the mouse at a time of their own choosing, which again produced a tone. On these trials however, participants were asked to report where the clock hand was when they heard the tone, not when they clicked the mouse. At the beginning of each block, participants completed five practice trials to familiarize themselves with the procedure. Practice trials were not included in the analysis.

## Results

For each participant, action and tone judgments that deviated more than 2.5 standard deviations from the mean judgment for a particular condition were excluded. This resulted in the removal of less than 1% of trials. Remaining action and tone judgment data were subjected to inferential statistical analysis.

### Calculating action, tone and total shifts

**Figure 2:**
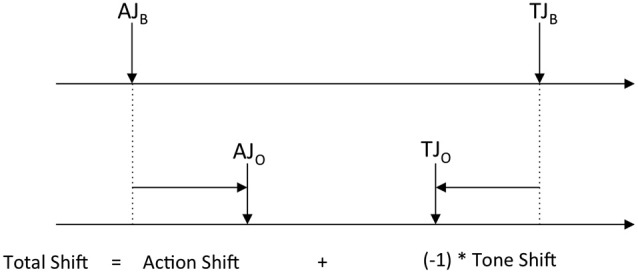
**Illustrates how action, tone, and overall degree of binding (i.e., total shift) are calculated**. AJ_B_: Action judgment in baseline condition, TJ_B_: Tone judgment in baseline condition. AJ_O_: Action judgment in operant condition, TJ_O_: Tone judgment in operant condition.

To determine perceptual shifts, we first calculated judgment errors by quantifying the difference between judgments of actions and tones compared to their veridical onset times, for both baseline and operant conditions. The difference between these judgment errors for baseline and operant conditions was taken as the perceived shift. In addition, the overall “degree of binding” (or “total shift”) was determined by calculating the extent to which the perceived times of actions and tones moved towards each other. This was calculated as: (Action shift) + (−1xTone shift) (see Figure 2).

### NPI score and agency

**Figure 3:**
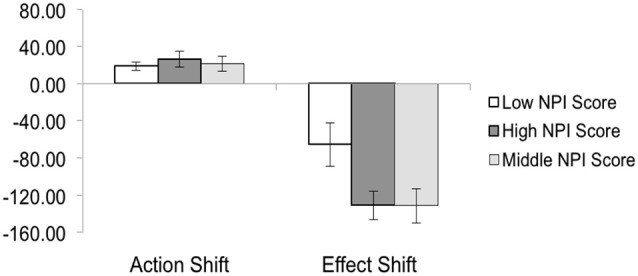
**The mean action shift and mean tone shift for the low, middle and high NPI score group**. Tone shift was significantly greater in the high and middle groups compared to the low group. Error bars are SEM. See text for statistics.

**Figure 4:**
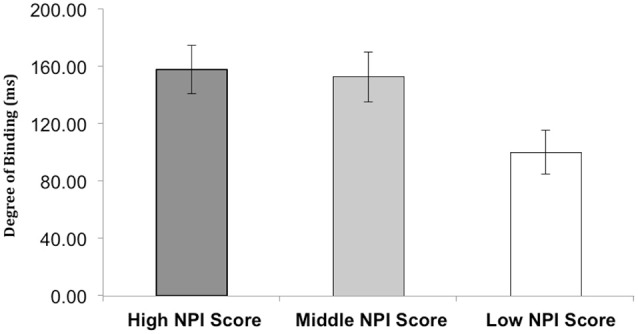
**The mean total degree of binding (in ms) for the low, middle, and high NPI score groups; the high and middle group showed significantly more binding than the low group**. Error bars are SEM. See text for statistics.

The three groups were classified as follows: High NPI score, who had scores greater than 21, Middle NPI scores who had scores between 11–17, and Low NPI scores, who had scores between 3–9. Participant binding data from the three groups were entered into three separate one-way ANOVAs for analysis. There was a main effect of group on Tone shift (*F*_(2,24)_ = 3.759, *p* < 0.05), as well as on Total shift (*F*_(2,24)_ = 3.643, *p* < 0.05). However there was no effect of group on Action shift (*F*_(2,24)_ = 0.319, *p* > 0.05). Follow up independent samples *t*-tests were run to investigate the difference in the degree of shift between High, Middle and Low groups (mean shift data for actions and effects are presented in Figure 3, overall binding data is presented in Figure 4).

### High vs. low NPI scores

Follow up independent samples *t*-tests were run to investigate the difference in the degree of shift between High and Low NPI score participants (mean shift data for actions and effects is presented in Figure 3, and overall binding is presented in Figure 4). The *t*-test revealed a significant difference for tone shifts (High: Mean = −130.97, SD = 46.25 < Low: Mean = −65.18, SD = 71.20, *t*_(16)_ = 2.325, *p* = 0.034) and overall binding (High: Mean = 157.40, SD = 51.08 > Low: Mean = 100.20, SD = 45.94, *t*_(16)_ = −2.498, *p* = 0.024), but not for action shifts (High: Mean = 26.36, SD = 26.73 < Low: Mean = 18.62, SD = 13.03, *t*_(15)_ = −0.304, *p* = 0.765).

### Low vs. middle NPI scores

The *t*-tests also revealed a significant difference for tone shifts between Middle and Low groups (Middle: Mean = −131.05, SD = 56.28 < Low: Mean = −65.18, SD = 71.20, *t*_(16)_ = 2.177, *p* = 0.045) (see Figure 3), and for overall degree of binding (Middle: Mean = 152.67, SD = 52.45 > Low: Mean = 100.20, SD = 45.94, *t*_(16)_ = −2.258, *p* = 0.038) (see Figure 4), but not action shift (Middle: Mean = 21.62, SD = 25.02 > Low: Mean = 18.62, SD = 13.03, *t*_(15)_ = −0.304, *p* = 0.765) (see Figure 3).

### Middle vs. high NPI scores

The High and Middle groups did not significantly differ on the action (Middle: Mean = 21.62, SD = 25.02 < High: Mean = 26.36, SD = 26.73, *t*_(16)_ = −0.388, *p* = 0.703) or effect shifts (Middle: Mean = −131.05, SD = 56.28 < High: Mean = −130.97, SD = 46.25, *t*_(16)_ = −0.003, *p* = 0.998) (see Figure 3), nor on overall binding (Middle: Mean = 152.67, SD = 52.45 < High: Mean = 157.40, SD = 51.08, *t*_(16)_ = −0.914, *p* = 0.849) (see Figure 4).

## Discussion

The current study investigated whether individuals who differ on their score on the NPI also show different patterns of intentional binding when making judgments about the onsets of voluntary actions and their effects. Given that narcissistic traits are associated with increased dominance motivation and behavior, an over-inflated sense of self-importance and a tendency to seek out social power as a means to maintain high social status, we hypothesized that those who scored higher on the NPI would exhibit a correspondingly greater degree of intentional binding.

Our prediction was borne out by the results. Individuals with higher NPI scores did indeed display greater levels of binding than those with low NPI scores, although the effect was entirely driven by shifts in perception of the tone. Interestingly the middle group displayed tone binding that was indistinguishable from the high group. This is most likely due to the fact that none of our participants scored anywhere near the maximum NPI score of 40. Thus, one limitation of the current initial study, is that our groups, although split on the basis of the range of NPI scores we obtained, did not include scores at the upper end of the NPI scale itself. Thus, high and middle levels of narcissism in our sample, perhaps corresponded to a single “moderate” group and may reflect healthy levels of narcissism (Maxwell et al., 2011). Indeed, it could be argued that, in the absence of obtaining scores right at the high end of the NPI scale, we are not dealing with narcissism at all in the current sample. However, we do have different ranges of NPI scores and to avoid mislabeling a moderate NPI score as a moderate level of narcissism, we simply refer to moderate NPI scores instead of middle and high narcissism, for the remainder of the discussion.

Not withstanding the lack of high NPI scores, our results show a clear difference in binding between individuals with moderate NPI scores and low NPI scores. Importantly the low group did contain scores as low as 3, and therefore our results are consistent with reduced agency for individuals at the low end of the NPI scale. Indeed, low scores on the NPI may be comorbid with other psychological characteristics such as low self-esteem, anxiety and/or depression, which have been shown to be related to a reduced sense of agency (Barlow, 1991; Keeton et al., 2008; Obhi et al., 2012, 2013). Despite the lack of very high NPI scores in the current study, overall, our results provide the first evidence that different scores on a personality trait are associated with differences in the degree of binding of effects to voluntary actions, and by extension, pre-reflective agentic experience.

Our results suggest that even moderate scores on the NPI might be linked to a stronger sense of agency and increased intentional binding for voluntary actions and outcomes, compared to lower levels of narcissism. Furthermore, while it is well known that narcissists often over-estimate their intelligence and their academic abilities (Robins and Beer, 2001; Campbell et al., 2002), among other things, it may be that those who score very low on the NPI may correspondingly under-estimate their abilities. Specifically, the decreased level of tone binding they display suggests that they may particularly under-estimate the degree of control they have over the outcomes that their actions produce. Given that low self-esteem has been linked to risk for depression (Orth et al., 2008), and that we recently showed that activating memories of depression reduces intentional binding (Obhi et al., 2013), one plausible explanation for the current pattern of data is indeed that individuals with low NPI scores are less psychologically “healthy” than their moderate scoring counterparts, and one consequence of this is that they have diminished agentic experience. Binding is an intriguing method for examining differences in the experience of voluntary action and further research is required to clarify the precise relationship between narcissism, psychological health and agency. This study represents an initial demonstration that such a relationship may exist, and is therefore worthy of further investigation. More generally, this study underscores that personality differences do impact the experience of voluntary action and thereby open up a new area of inquiry for researchers working on volitional action.

A noteworthy aspect of the current results is that the action-outcome complex that was employed in the experiment was arbitrary (a key press followed by an auditory tone) and did not involve control over other social agents. An obvious and potentially illuminating extension of this work involves comparing binding for arbitrary action outcomes such as lights and tones, with social outcomes such as “winning”, “losing” or influencing the actions of another individual (see Obhi et al., 2012 for a similar approach). Indeed, previous authors have commented on the tendency for narcissists to subjugate others in their social environment and “use” them in the service of their own goals (Dimaggio et al., 2002). One prediction is that such manipulations would increase the influence of narcissism on binding.

Future work might consider investigating intentional binding in patients with clinical narcissism. Since NPD has been more recently associated with a deeply held sense of low self-esteem (Ronningstam, 2011b), the intentional binding effect in patients may mimic those who had abnormally low scores on the NPI. Unlike trait anxiety, which is highly correlated to anxiety disorder (Grupe and Nitschke, 2013), narcissism as a personality trait (measured on the NPI) is often not well correlated to the full-blown experience of narcissistic disorder. Thus, non-patients tend to score lower on the NPI than healthy participants, and higher scoring on the NPI by narcissists could simply be a function of response bias (John and Robins, 1994; Pincus and Lukowitsky, 2010). Furthermore, some researchers have suggested that the sense of agency in NPD is more vulnerable and experiences more fluctuations than that in non-narcissists. In view of this it would be beneficial to test clinical narcissists on the intentional binding task and to measure how the degree of binding changes after receiving criticism, or other types of feedback. Changes in binding, as a function of the social circumstance, may explain the variability experienced in self-agency by narcissists, and can further aid to explain why narcissists tend to shift between different periods of high and low functioning (Ronningstam, 2013; Ronningstam and Baskin-Sommers, 2013). Again though, we underline that in the current study we simply measured NPI scores and determined whether different scores were associated with differences in binding. We likely did not have “real” narcissists in our sample and thus our ideas for future work on clinical samples must be treated as speculative.

There has been considerable research interest in intentional binding since it was first reported in 2002 (Haggard et al., 2002; Moore and Obhi, 2012). Out of this research, strong support for the notion that preparatory and predictive, processes play an important role in binding, and particularly tone binding, has emerged. The comparator model is an influential model of motor control that posits interaction between a prediction of the sensory consequences of pending movement and the actual sensory consequences of the movement (Blakemore et al., 1999). This comparator model has been invoked in the study of agency and it has been shown that when accurate prediction is not possible, the sense of agency, and intentional binding is reduced (Haggard and Clark, 2003; Tsakiris and Haggard, 2003). Specifically, an influential model of agency proposes that when the prediction of the sensory consequences of an action and the actual sensory consequences match, agency is experienced, whereas, when they do not match, the action is not attributed to the self (Blakemore et al., 2002).

The supplementary motor area (SMA) is thought to be a key region involved in action preparation and prediction as well as the conscious experience of motor intentions (Fried et al., 1991; Makoshi et al., 2011; Moore and Obhi, 2012). Interestingly, theta burst TMS over the pre-supplementary motor area (pre-SMA) has been shown to reduce tone binding in neurologically normal participants, apparently confirming a key role for the pre-SMA in subjective experience of action effects (Moore et al., 2010). More generally, prediction has been purported as a fundamental brain process that enables successful navigation of the environment, both physical and social (e.g., Bubic et al., 2010). Taken together these studies lend support to the notion that premotor processing is strongly tied the phenomenology of action and effect binding. Thus, it is possible that individuals with low NPI scores experience lower levels of motor preparation or differ in their predictive processing compared to those with moderate NPI scores.

In addition to the possible role of prediction, it has also been shown that the binding of outcomes back toward actions can be the result of inferential processes that take into account the probability of actions producing effects. In this sense, binding is brought about not by prediction, but by a postdictive process (e.g., Wegner, 2002). For example, when additional effects occur that are not linked to actions, effect binding is reduced compared to when these additional non-action related effects do not occur (Moore et al., 2009). The manner in which the pre-SMA might contribute to postdictive processes remains to be elucidated, and given current knowledge of pre-SMA function, a predictive influence on binding may be more likely. Another important finding that fits well with our current results is that when an agent has a strong prior belief that they will cause an outcome, they show stronger effect binding (Desantis et al., 2011). In our experimental context, this result suggests that those with low NPI scores may have a chronically weak belief in themselves as causal agents, whereas those with moderate NPI scores have a stronger chronic belief in themselves as causal agents. As Desantis et al. (2011) suggested this difference in the strength of a priori beliefs could affect the reliability that the brain places on predictions of a forward model. Future work should consider this possibility further.

Future work could also address these possibilities by employing neuroimaging to assess the level of preparatory activity in the SMA (among other areas) in clinical narcissists and by manipulating the ability to predict sensory consequences of actions (by varying the probability of an effect occurring, for example). The suggestion that differences in trait narcissism may be linked with differences in sensorimotor prediction is, to our knowledge, relatively novel, and warrants further investigation.

The initial study we present here suffers from several limitations, some of which have been mentioned above. First, our sample was smaller than ideal and did not contain any individuals who scored above 33/40 on the NPI. This may have reduced differences in between our high and middle group in particular, which might account for the similar levels of binding displayed by these groups. Thus, one important follow-up study will be to recruit individuals whose scores fall along the full range of the NPI scale with at least 12 participants per group, and it must be underlined that this study cannot directly shed light on how clinical narcissists might manifest in intentional binding tasks. Second, we did not assess other psychological characteristics that may be correlated with different levels of narcissism (e.g., self-esteem). Another possibility is that different facets of narcissism are associated with different facets of cognition, including agentic dominance or causal reasoning; involving adaptive and/or maladaptive outcomes (Vonk et al., 2013). Measuring binding in relation to the subscales of the NPI may shed further light on variability in perceptual shifts within the three groups; although it is still unclear how many and to what extent the factors in these subscales exist (Ackerman et al., 2011). We also had a sample that was heavily biased towards females, who may experience narcissism differently, as gender differences have been described in other mental illnesses or trait characteristics (Greaves-Lord et al., 2010; McLean et al., 2011).

In sum, we report seminal results demonstrating a relationship between scores on a personality scale, the NPI and intentional binding. These results show that different scores on the NPI are associated with changes in the subjective experience of sensory effects produced by voluntary actions. Thus, to the extent that binding indexes agency, our results also provide evidence that low-level, pre-reflective agency is lower in individuals who score lower on the NPI compared to their counterparts who have moderate scores on the NPI. In future studies, measuring the degree of intentional binding in clinically diagnosed narcissists could provide insight to their inner most state: are they overly agentic, confident, and self loving; or are they over compensating for feelings of worthlessness, low self-esteem and lack of control (see Bosson et al., 2008)? Indeed, the development of agency measures that circumvent self-presentational biases could eventually be valuable in the diagnosis of personality disorders and may be relevant to new ideas regarding levels of functioning and assessment criteria in the diagnostic and statistical manual of mental disorders (DSM-5; see Skodol, 2012). These are questions that would be hard to address via the use of more traditional explicit measures that are hampered by self-presentation issues. Finally, the present work underscores the importance of assessing individual/personality differences in the performance and experience of volitional action, which allows the field to move beyond reliance on group level data.

## Conflict of interest statement

The authors declare that the research was conducted in the absence of any commercial or financial relationships that could be construed as a potential conflict of interest.

## References

[B1] Ackerman R. A. Witt E. A. Donnellan M. B. Trzesniewski K. H. Robins R. W. Kashy D. A. (2011). What does the narcissistic personality inventory really measure? Assessment 18, 67–87. 10.1177/1073191110382845 20876550

[B2] Barlow D. H. (1991). “The nature of anxiety: anxiety, depression and emotional disorders,” in Chronic Anxiety: Generalized Anxiety Disorder and Mixed Anxiety Depression, eds Rapee R. M. Barlow D. H. (New York: Guilford Press), 1–28.

[B3] Baumeister R. F. Bushman B. J. Campbell W. K. (2000). Self-esteem, narcissism and aggression: does violence result from low self-esteem or from threatened egotism? Curr. Dir. Psychol. Sci. 9, 26–29 10.1111/1467-8721.00053

[B4] Blakemore S. Frith C. D. Wolpert D. M. (1999). Spatio-temporal prediction modulates the perception of self-produced stimuli. J. Cogn. Neurosci. 11, 551–559. 10.1162/089892999563607 10511643

[B5] Blakemore S. Wolpert D. M. Frith C. D. (2002). Abnormalities in the awareness of action. Trends Cogn. Sci. 6, 237–242. 10.1016/s1364-6613(02)01907-1 12039604

[B6] Bosson J. K. Lakey C. E. Campbell W. K. Zeigler-Hill V. Jordan C. H. Kernis M. H. (2008). Untangling the links between narcissism and self-esteem: a theoretical and empirical review. Soc. Personal. Psychol. Compass. 2, 1415–1439 10.1111/j.1751-9004.2008.00089.x

[B7] Bubic A. von Cramon D. Y. Schubotz R. I. (2010). Prediction, cognition and the brain. Front. Hum. Neurosci. 4:25. 10.3389/fnhum.2010.00025 20631856 PMC2904053

[B8] Buehner M. J. (2012). Understanding the past, predicting the future: causation, not intentional action, is the root of temporal binding. Psychol. Sci. 23, 1490–1497. 10.1177/0956797612444612 23104679

[B9] Campbell W. K. Bosson J. K. Goheen T. W. Lakey C. E. Kernis M. H. (2007). Do narcissists dislike themselves “deep down inside?”. Psychol. Sci. 18, 227–229. 10.1111/j.1467-9280.2007.01880.x 17444918

[B12] Campbell W. K. Rudich E. A. Sedikides C. (2002). Narcissism, self-esteem and the positivity of self-views: two portraits of self-love. Pers. Soc. Psychol. Bull. 28, 358–368 10.1177/0146167202286007

[B64] Desantis A. Roussel C. Waszak F. (2011). On the influence of causal beliefs on the feeling of agency. Conscious. Cogn. 20, 1211–1220. 10.1016/j.concog.2011.02.012 21396831

[B13] Dimaggio G. Fiore D. Lysaker P. H. Petrilli D. Salvatore G. Semerari A.. (2006). Early narcissistic transference patterns: an exploratory single case study from the perspective of dialogical self theory. Psychol. Psychother. 79, 495–516. 10.1348/147608305x63089 17312867

[B14] Dimaggio G. Nicolo G. Fiore D. Centenero E. Semerari A. Carcione A.. (2008). States of minds in narcissistic personality disorder: three psychotherapies analyzed using the grid of problematic states. Psychother. Res. 18, 466–480. 10.1080/10503300701881877 18815998

[B15] Dimaggio G. Semerari A. Falcone M. Nicolò G. Carcione A. Procacci M. (2002). Metacognition, states of mind, cognitive biases and interpersonal cycles: proposal for an integrated narcissism model. J. Psychother. Integr. 12, 421–451 10.1037//1053-0479.12.4.421

[B16] Fried I. Katz A. McCarthy G. Sass K. J. Williamson P. Spencer S. S.. (1991). Functional organization of human supplementary motor cortex studied by electrical stimulation. J. Neurosci. 11, 3656–3666. 1941101 10.1523/JNEUROSCI.11-11-03656.1991 PMC6575551

[B17] Gallagher S. (2012). Multiple aspects in the sense of agency. New Ideas Psychol. 30, 15–31 10.1016/j.newideapsych.2010.03.003

[B19] Greaves-Lord K. Tulen J. Dietrich A. Sondeijker F. Van Roon A. Oldehinkel A.. (2010). Reduced autonomic flexibility as a predictor for future anxiety in girls from the general population: the TRAILS study. Psychiatry Res. 179, 187–193. 10.1016/j.psychres.2009.04.014 20483486

[B20] Grupe D. W. Nitschke J. B. (2013). Uncertainty and anticipation in anxiety: an integrated neurobiological and psychological perspective. Nat. Rev. Neurosci. 14, 488–501. 10.1038/nrn3524 23783199 PMC4276319

[B21] Haggard P. (2001). The psychology of action. Br. J. Psychol. 92, 113–128. 10.1348/000712601162121 11256760

[B22] Haggard P. Clark S. (2003). Intentional action: conscious experience and neural prediction. Conscious. Cogn. 12, 695–707. 10.1016/s1053-8100(03)00052-7 14656511

[B23] Haggard P. Clark S. Kalogeras J. (2002). Voluntary action and conscious awareness. Nat. Neurosci. 5, 382–385. 10.1038/nn827 11896397

[B24] Haggard P. Martin F. Taylor-Clarke M. Jeannerod M. Franck N. (2003). Awareness of action in schizophrenia. Neuroreport 14, 1081–1085. 10.1097/00001756-200305230-00035 12802207

[B27] John O. P. Robins R. W. (1994). Accuracy and bias in self-perception: individual differences in self-enhancement and the role of narcissism. J. Pers. Soc. Psychol. 66, 206–219. 10.1037//0022-3514.66.1.206 8126650

[B28] Keeton C. P. Perry-Jenkins M. Sayer A. G. (2008). Sense of control predicts depressive and anxious symptoms across the transition to parenthood. J. Fam. Psychol. 22, 212–221. 10.1037/0893-3200.22.2.212 18410208 PMC2834184

[B29] Kirkpatrick L. A. Waugh C. E. Valencia A. Webster G. D. (2002). The functional domain specificity of self-esteem and the differential prediction of aggression. J. Pers. Soc. Psychol. 82, 756–767. 10.1037//0022-3514.82.5.756 12003475

[B61] Kohut H. (1971). The Analysis of the Self: A Systematic Approach to the Psychoanalytic Treatment of Narcissistic Personality Disorders. Chicago, IL: University of Chicago Press Available online at: http://search.proquest.com/docview/622249253?accountid=12347

[B30] Kohut H. (1977). The Restoration of the Self. Chicago, IL: University of Chicago Press.

[B32] Kranick S. M. Campen C. J. Kasner S. E. Kessler S. K. Zimmerman R. A. Lustig R. A.. (2013). Headache as a risk factor for neurovascular events in pediatric brain tumor patients. Neurology 80, 1452–1456. 10.1212/wnl.0b013e31828cf81e 23486881 PMC3662360

[B33] Makoshi Z. Kroliczak G. van Donkelaar P. (2011). Human supplementary motor area contribution to predictive motor planning. J. Mot. Behav. 43, 303–309. 10.1080/00222895.2011.584085 21732868

[B34] Maxwell K. Donnellan M. B. Hopwood C. J. Ackerman R. A. (2011). The two faces of narcissus? An empirical comparison of the narcissistic personality inventory and the pathological narcissism inventory. Pers. Individ. Dif. 50, 577–582 10.1016/j.paid.2010.11.031

[B35] McLean C. P. Asnaani A. Litz B. T. Hofmann S. G. (2011). Gender differences in anxiety disorders: prevalence, course of illness, comorbidity and burden of illness. J. Psychiatr. Res. 45, 1027–1035. 10.1016/j.jpsychires.2011.03.006 21439576 PMC3135672

[B37] Moore J. W. Lagnado D. Deal D. C. Haggard P. (2009). Feelings of control: contingency determines experience of action. Cognition 110, 279–283. 10.1016/j.cognition.2008.11.006 19110240

[B38] Moore J. W. Obhi S. S. (2012). Intentional binding and the sense of agency: a review. Conscious. Cogn. 21, 546–561. 10.1016/j.concog.2011.12.002 22240158

[B39] Moore J. W. Ruge D. Wenke D. Rothwell J. Haggard P. (2010). Disrupting the experience of control in the human brain: pre-supplementary motor area contributes to the sense of agency. Proc. Biol. Sci. 277, 2503–2509. 10.1098/rspb.2010.0404 20375048 PMC2894930

[B40] Moretto G. Walsh E. Haggard P. (2011). Experience of agency and sense of responsibility. Conscious. Cogn. 20, 1847–1854. 10.1016/j.concog.2011.08.014 21920776

[B41] Morf C. C. Rhodewalt F. (2001). Unraveling the paradoxes of narcissism: a dynamic self-regulatory processing model. Psychol. Inq. 12, 177–196 10.1207/s15327965pli1204_1

[B43] Obhi S. S. Goodale M. A. (2005). Bimanual interference in rapid discrete movements is task specific and occurs at multiple levels of processing. J. Neurophysiol. 94, 1861–1868. 10.1152/jn.00320.2005 15917318

[B44] Obhi S. S. Hall P. (2011). Sense of agency and intentional binding in joint action. Exp. Brain Res. 211, 655–662. 10.1007/s00221-011-2675-2 21503647

[B45] Obhi S. S. Sebanz N. (2011). Moving together: toward understanding the mechanisms of joint action. Exp. Brain Res. 211, 329–336. 10.1007/s00221-011-2721-0 21573952

[B46] Obhi S. S. Swiderski K. M. Brubacher S. P. (2012). Induced power changes the sense of agency. Conscious. Cogn. 21, 1547–1550. 10.1016/j.concog.2012.06.008 22781399

[B47] Obhi S. S. Swiderski K. M. Farquhar R. (2013). Activating memories of depression alters the experience of voluntary action. Exp. Brain Res. 229, 497–506. 10.1007/s00221-012-3372-5 23247470

[B48] Orth U. Robins R. W. Roberts B. W. (2008). Low self-esteem prospectively predicts depression in adolescence and young adulthood. J. Pers. Soc. Psychol. 95, 695–708. 10.1037/0022-3514.95.3.695 18729703

[B49] Pincus A. L. Lukowitsky M. R. (2010). Pathological narcissism and narcissistic personality disorder. Annu. Rev. Clin. Psychol. 6, 421–446. 10.1146/annurev.clinpsy.121208.131215 20001728

[B50] Raskin R. Novacek J. Hogan R. (1991). Narcissistic self-esteem management. J. Pers. Soc. Psychol. 60, 911–918 10.1037/0022-3514.60.6.911

[B52] Robins R. W. Beer J. S. (2001). Positive illusions about the self: short-term benefits and long-term costs. J. Pers. Soc. Psychol. 80, 340–352. 10.1037/0022-3514.80.2.340 11220450

[B53] Ronningstam E. (2011a). Complex case: narcissistic personality disorder. Personal. Ment. Health 5, 222–227 10.1002/pmh.172

[B62] Ronningstam E. (2011b). Narcissistic personality disorder: a clinical perspective. J. Psychiatr. Pract. 17, 89–99. 10.1097/01.pra.0000396060.67150.40 21430487

[B54] Ronningstam E. (2013). An update on narcissistic personality disorder. Curr. Opin. Psychiatry 26, 102–106. 10.1097/YCO.0b013e328359979c 23187086

[B55] Ronningstam E. Baskin-Sommers A. R. (2013). Fear and decision-making in narcissistic personality disorder-a link between psychoanalysis and neuroscience. Dialogues Clin. Neurosci. 15, 191–201. 24174893 10.31887/DCNS.2013.15.2/eronningstam PMC3811090

[B56] Rosenbaum D. (1991). Human Motor Control. San Diego, California: Academic Press.

[B57] Skodol A. E. (2012). Personality disorders in DSM-5. Annu. Rev. Clin. Psychol. 8, 317–344. 10.1146/annurev-clinpsy-032511-143131 22458868

[B58] Tsakiris M. Haggard P. (2003). Awareness of somatic events associated with a voluntary action. Exp. Brain Res. 149, 439–446. 10.1007/s00221-003-1386-8 12677324

[B59] Vonk J. Zeigler-Hill V. Mayhew P. Mercer S. (2013). Mirror, mirror on the wall, which form of narcissist knows self and others best of all? Pers. Individ. Dif. 54, 396–401 10.1016/j.paid.2012.10.010

[B63] Wegner D. M. (2002). The Illusion of Conscious Will. Cambridge, MA: MIT Press Available online at: http://search.proquest.com/docview/619864394?accountid=12347

